# Ranking-based hierarchical random mutation in differential evolution

**DOI:** 10.1371/journal.pone.0245887

**Published:** 2021-02-04

**Authors:** Xuxu Zhong, Meijun Duan, Peng Cheng

**Affiliations:** 1 National Key Laboratory of Fundamental Science on Synthetic Vision, Sichuan University, Chengdu, China; 2 School of Computer and Software Engineering, Xihua University, Chengdu, China; 3 School of Aeronautics and Astronautics, Sichuan University, Chengdu, China; Max-Planck-Institut fur Mathematik in den Naturwissenschaften, GERMANY

## Abstract

In order to improve the performance of differential evolution (DE), this paper proposes a ranking-based hierarchical random mutation in differential evolution (abbreviated as RHRMDE), in which two improvements are presented. First, RHRMDE introduces a hierarchical random mutation mechanism to apply the classic “DE/rand/1” and its variant on the non-inferior and inferior group determined by the fitness value. The non-inferior group employs the traditional mutation operator “DE/rand/1” with global and random characteristics, which increases the global exploration ability and population diversity. The inferior group uses the improved mutation operator “DE/rand/1” with elite and random characteristics, which enhances the local exploitation ability and convergence speed. Second, the control parameter adaptation of RHRMDE not only considers the complexity differences of various problems but also takes individual differences into account. The proposed RHRMDE is compared with five DE variants and five non-DE algorithms on 32 universal benchmark functions, and the results show that the RHRMDE is superior over the compared algorithms.

## 1 Introduction

In the field of problem optimization, metaheuristic algorithms have been paid more attention and researched by many scholars because of their fast calculation capabilities based on computer simulation and no need for complicated mathematical formula derivation, so they have developed rapidly. In addition to classic Genetic Algorithm (GA) [1], Simulated Annealing (SA) [2], Particle Swarm Optimization (PSO) [3], Differential Evolution (DE) [4], Artificial Bee Colony (ABC) [5], Firefly Algorithm (FA) [6], etc., many new metaheuristic algorithms are also emerging, such as Grey Wolf Optimizer (GWO) [7], Whale Optimization Algorithm (WOA) [8], Monkey King Evolution (MKE) [9], Marine Predators Algorithm (MPA) [10], Gaining Sharing Knowledge based Algorithm (GSK) [11] and Equilibrium Optimizer (EO) [12], etc. Differential evolution (DE) is a branch of metaheuristic algorithms based on population for optimization, which performs parallel search and obtains information in the search space through the differences among individuals in the population. Because of its high efficiency, simplicity and easy operation, DE has been successfully applied in many fields [13–18]. However, like other metaheuristic algorithms, when solving some complex optimization problems, DE is also prone to premature convergence and fall into a local optimal solution [19, 20]. To overcome these shortcomings and improve the convergence speed and stability of DE, many scholars have carried out a lot of research work, most of which based on control parameters and mutation strategies [21].

First, many researchers focused on the optimization of DE control parameters, such as population size *NP*, scaling factor *F* and crossover probability *CR*. Liu and Lampinen [22] proposed a fuzzy adaptive differential evolution (FADE), in which individuals successfully entered the next generation and their fitness values were used as inputs to a fuzzy logic controller to adjust *F* and *CR*. Noman [23] proposed an adaptive differential evolution Algorithm (aDE), which compared the fitness of the offspring individual with the average fitness value of the parent population. If the former was smaller, *F* and *CR* would remain unchanged. Otherwise, they would be randomly selected in the interval [0.1, 1.0] and [0.0, 1.0] respectively. Ghosh [24] proposed an improved differential evolution algorithm with fitness-based adaptation of the control parameters (FiADE), in which the adjustment of *F* and *CR* was based on the objective function value of individuals in the population. Sarker [25] proposed a DE with dynamic Parameters Selection algorithm (DE-DPS), in which *NP*, *F* and *CR* were dynamically selected from corresponding candidate sets according to their historical performance for adaptive looping. Tanabe and Fukunaga [26] proposed success-history based adaptive differential evolution (SHADE), which saved the control parameters when the evolution is successful in the historical memories *M*_*F*_, and *M*_*CR*_ to guide the selection of future control parameter values. Based on SHADE [26], Viktorin [27] adjusted *F* and *CR* based on the Euclidean distance between the trial individual and the original individual. Draa [28] proposed a sinusoidal differential evolution (SinDE), the adjustment of *F* and *CR* in which was based on the use of a new sinusoidal framework.

Second, some scholars paid much attention to the improvement of DE’s mutation strategy. Qin et al. [29] proposed a self-adaptive DE (SaDE), which adopted four mutation strategies to generate trial vectors. The selection of mutation strategies would be affected by the performance of these four strategies. Zhang and Sanderson [30] proposed an adaptive differential evolution with optional external archive algorithm (JADE), which introduced a new mutation strategy “DE/current−to−pbest/1”. In contrast to the classic mutation strategy “DE/current−to−best/1”, the best individual in the classic was replaced by an individual randomly selected from the top *P*%. Gong and Cai [31] proposed an improved adaptive differential evolution with crossover rate repairing technique and ranking-based mutation called Rcr-IJADE, which was an enhanced version of JADE [30]. Epitropakis [32] proposed a proximity-based mutation algorithmic scheme for DE/rand/1. Mallipeddi [33] proposed a differential evolution algorithm with ensemble of parameters and mutation strategies (EPSDE). During the entire evolution process, different mutation strategy pools coexisted with the value pools of each control parameter and competed with each other to produce offspring. Fan and Yan [34] presented an adaptive differential evolution algorithm with discrete mutation control parameters (DMPSADE), which assigned mutation strategies to individuals based on the roulette wheel. Tang [35] proposed a differential evolution with an individual-dependent mechanism (IDE), which contained four mutation operators used in different stages. Wu [36] proposed a differential evolution with multi-population based ensemble of mutation strategies (MPEDE), which partitioned the population dynamics into three indicator subpopulations and one reward subpopulation. Each indicator subpopulation had its own mutation strategy, and the reward subpopulation was applied to the mutation strategy that had performed best so far. Duan [37] proposed a differential evolution algorithm with dual preferred learning mutation (DPLDE), which selected the random mutation vector based on the fitness value and the Euclidean distance between individuals, and then adjusted the mutation strategy according to the randomly selected individual, the optimal individual and the target individual. Mohamed AW and Mohamed AK [38] proposed an adaptive guided differential evolution algorithm with novel mutation for numerical optimization (AGDE), in which individuals were randomly selected from the best 100*P*% and the worst 100*P*% of the population and their difference vectors were used as the disturbance in the mutation operation. Besides, the base vector of the mutation operator was randomly selected from the remaining [*NP*−2(100*P*%)] individuals. Wei [39] proposed a random perturbation modified differential evolution algorithm (PRMDE), which contained a new "DE/M_pBest−best/1" mutation operator, and the population mutation operation would be implemented based on this proposed mutation operator or the classic mutation operator "DE/best/1". Wang [40] proposed a self-adaptive mutation differential evolution algorithm based on particle swarm optimization (DEPSO), in which a selection probability determined whether an individual would apply “DE/e−rand/1” mutation strategy or PSO mutation strategy.

In order to overcome the problem of premature convergence on DE and improve its performance, a differential evolution with ranking-based hierarchical random mutation in (RHRMDE) is proposed in this paper. Firstly, a hierarchical random mutation mechanism is designed. The upper layer of the model corresponds to a random global mutation strategy, which is applied by superior (*NP*−*NWP*) individuals in the population sorted by fitness values. *NP* and *NWP* represent the population size and the inferior group size, respectively. At the same time, the lower layer of the model designs a random elite mutation strategy, using individuals randomly selected from superior *NWP* individuals to guide the evolution of *NWP* inferiors. Secondly, in the adjustment of the control parameters, the complexity differences of diverse problems and individual differences are considered, as well as the evolutionary status of individuals in the previous generation. In addition, the sensitivity of the inferior group size *NWP* has been analyzed in this paper. Furthermore, the performance of the proposed RHRMDE is verified on 32 benchmark functions by comparing with five DE variants and five non-DE algorithms, and the experiment results demonstrate that RHRMDE outperforms the compared algorithms.

The rest of this paper is structured as follows. Section 2 gives the classic DE algorithm briefly. Section 3 introduces details of the proposed RHRMDE algorithm. The experimental results compared with five DE variants and five non-DE algorithms are reported and analyzed in Section 4. Section 5 draws the conclusions of this work and offers the future research direction.

## 2 The classic differential evolution algorithm

In DE, the population $P^{G}=\{X_{1}^{G},X_{2}^{G},\cdots ,X_{NP}^{G}\}$ at generation *G* consists of *NP* individuals $X_{i}^{G}=\{x_{i,1}^{G},x_{i,2}^{G},\cdots ,x_{i,D}^{G}\}$, *i* = 1,2,⋯,*NP*, all of which has *D* dimensions. After initialization, the entire population generates the corresponding offspring through loop steps of mutation, crossover, selection until certain termination conditions are met.

### 2.1 Initialization

The *j*th component of the *i*th individual in the initial population can be expressed as:

$$x_{i,j}^{0}=x_{i,j}^{L}+{rand}_{j}*(x_{i,j}^{U}-x_{i,j}^{L})$$ (1)

where $x_{i,j}^{U}$ and $x_{i,j}^{L}$ are the upper and lower bounds of the *j*th dimension of the *i*th individual. *rand*_*j*_ returns a random number distributed between 0 and 1, *j* indicates that each component of the individual generates a new random number.

### 2.2 Mutation

After initialization, for each individual $X_{i}^{G}$, a mutation individual $V_{i}^{G+1}=\{v_{i,1}^{G+1},v_{i,2}^{G+1},\cdots ,v_{i,D}^{G+1}\}$ is generated through mutation operation. The classic mutation operator “DE/rand/1” can be expressed as:

$$V_{i}^{G+1}=X_{r1}^{G}+F\cdot \left(X_{r2}^{G}-X_{r3}^{G}\right)$$ (2)

where *F* is a scaling factor, *r*1, *r*2, *r*3 are randomly chosen indices within [1, *NP*] and are different from the current index *i*, that is, *r*1≠*r*2≠*r*3≠*i*.

### 2.3 Crossover

Next, for the target individual $X_{i}^{G}$ and its associated mutation individual $V_{i}^{G+1}$, a trial individual $U_{i}^{G+1}=\{u_{i,1}^{G+1},u_{i,2}^{G+1},\cdots ,u_{i,D}^{G+1}\}$ is produced by using binomial crossover.

$$u_{i,j}^{G+1}=\{\begin{array}{ll} v_{i,j}^{G+1} & {\mathrm{if}\;\mathrm{rand}}_{j}\leq CR\;\mathrm{or}\;j=j_{\mathrm{rand}}\\ x_{i,j}^{G} & \mathrm{otherwise} \end{array}\;j=1,2,\cdots ,D$$ (3)

where *CR*(∈[0,1]) determines the contribution of the mutation individual to the trial individual. *j*_*rand*_ denotes a random integer in the range of [1, *D*], which guarantees that at least one component (*j*_*rand*_) in the trial individual comes from the mutation individual.

### 2.4 Selection

After that, the selection step is executed: between the trial individual and the target individual, the one with the smaller fitness value will be accepted. In other words, if $f(U_{i,j}^{G+1})\leq f(X_{i}^{G})$, the trial individual $U_{i,j}^{G+1}$ will become the offspring individual $X_{i}^{G+1}$, otherwise the target individual $X_{i}^{G}$ will be retained.

$$X_{i}^{G+1}=\{\begin{array}{l} U_{i}^{G+1}\;if\;f\left(U_{i}^{G+1}\right)\leq f\left(X_{i}^{G}\right)\\ X_{i}^{G}\;otherwise \end{array}$$ (4)

## 3 RHRMDE algorithm

### 3.1 Hierarchical mutation mechanism

In RHRMDE, all individuals in the current population are sorted in descending order according to their fitness value, resulting in the new population as follows: $P^{G}=\{X_{best}^{G},X_{2}^{G},\cdots ,X_{NP-1}^{G},X_{worst}^{G}\}$, where $X_{best}^{G}$ is $X_{1}^{G}$ with the maximum fitness value; $X_{worst}^{G}$ is $X_{NP}^{G}$ with the minimum fitness value. Then the ranked population *P*^*G*^ is divided into two groups: the non-inferior group composed of the former (*NP*−*NWP*) individuals and the inferior group composed of the latter *NWP*. On this basis, a two-layer random mutation mechanism is adopted.

#### 3.1.1 The random global mutation strategy

Due to its random characteristic, the classic “DE/rand/1” has stronger global search capability and is applied to the non-inferior group at the individual level:

$$V_{i}^{G+1}=X_{r1}^{G}+F_{i}\cdot \left(X_{r2}^{G}-X_{r3}^{G}\right)$$ (5)

where *i* = {1,2,⋯,(*NP*−*NWP*)}, *r*1,*r*2,*r*3 differ from each other and range in the set {1,2,⋯,*NP*}. However, just because of the global and random properties, the classic mutation operator does not make full use of the information contained in superior individuals, which is beneficial for population evolution and convergence [30, 31, 38–41]. Then an improved version of the classic mutation strategy is proposed and applied to the “elite” level.

#### 3.1.2 The random elite mutation strategy

In this strategy, elite individuals guide the evolution of the inferior group. Meanwhile, for the purpose of accelerating convergence, a weighting factor is presented:

$$V_{i}^{G+1}=W_{i}\cdot X_{rp1}^{G}+F_{i}\cdot \left(X_{rp2}^{G}-X_{rp3}^{G}\right)$$ (6)

where *i* = {(*NP*−*NWP*+1),⋯,*NP*}, *W*_*i*_(∈[0,1]) is the weighting factor, $X_{rp1}^{G},$ $X_{rp2}^{G}$ and $X_{rp3}^{G}$ are elite individuals randomly selected from the former *NWP* individuals $\left\{X_{best}^{G},X_{2}^{G},\cdots ,X_{NWP}^{G}\right\}$. That is to say, *rp*1,*rp*2,*rp*3 range in the set {1,2,⋯,*NWP*} and *rp*1≠*rp*2≠*rp*3. In section 3.2, *F*_*i*_ and *W*_*i*_ will be discussed in more detail.

### 3.2 Control parameter adaptation

In the classic DE, control parameters are the same for all individuals, which have been applied in the individual level by many researchers later. Unlike most studies, the adaptation of control parameters (*F*, *CR*, *W*) in RHRMDE takes into account the complexity differences of various problems and individual differences.

#### 3.2.1 The scaling factor *F* adaptation

Due to control the size of the searching area around the base individual, the scaling factor *F* is one parameter used to balance the tradeoff between global and local search in RHRMDE. The scaling factor *F* is adjusted for the following purposes. Firstly, since the elite individuals in RHRMDE guide the evolution direction of the entire population, if a better individual can have more global search generations in the solution space, it is more likely to find a better solution, thereby reducing the probability of the population falling into local optima, so the index *i* representing individual differences is added to the adjustment of *F*. Secondly, for a simple optimization problem, it may take only a few dozen evolutionary generations to come up with a solution. However, for a complex optimization problem, it may not get close to the correct solution after hundreds of generations, but has already converged and fallen into the local optimal. Therefore, a factor related to the maximum generation number should be considered when calculating *F*, so that the change curves of F under different maximum generation numbers will not differ too much. Thirdly, using the evolutionary state of the previous generation to intervene in the adjustment of *F* helps to get rid of local optimality. Therefore, if evolution of the *ith* individual in the previous generation fails, the *F*_*i*_ of the *ith* individual in the current ranked population will be assigned a random value.

$$F_{i}=\left\{ \begin{array}{l} 0.9^{5∙i∙\frac{G}{G_{max}}},\;if\;{bs}_{i}^{G-1}=1\\ {rand}_{i}\;,\;if\;{bs}_{i}^{G-1}=0 \end{array}\right.$$ (7)

where *G* and *G*_*max*_ denote the current and the maximum generation number respectively. ${bs}_{i}^{G-1}$ is a binary variable that records whether the *ith* individual in the previous generation has evolved successfully. If the evolution is successful, then ${bs}_{i}^{G-1}=1$; otherwise, ${bs}_{i}^{G-1}=0.$ *rand*_*i*_ denotes a uniform random variable in the interval [0,1]. The random setting of *F*_*i*_ can improve the exploration ability by increasing population diversity.

**Fig 1:**
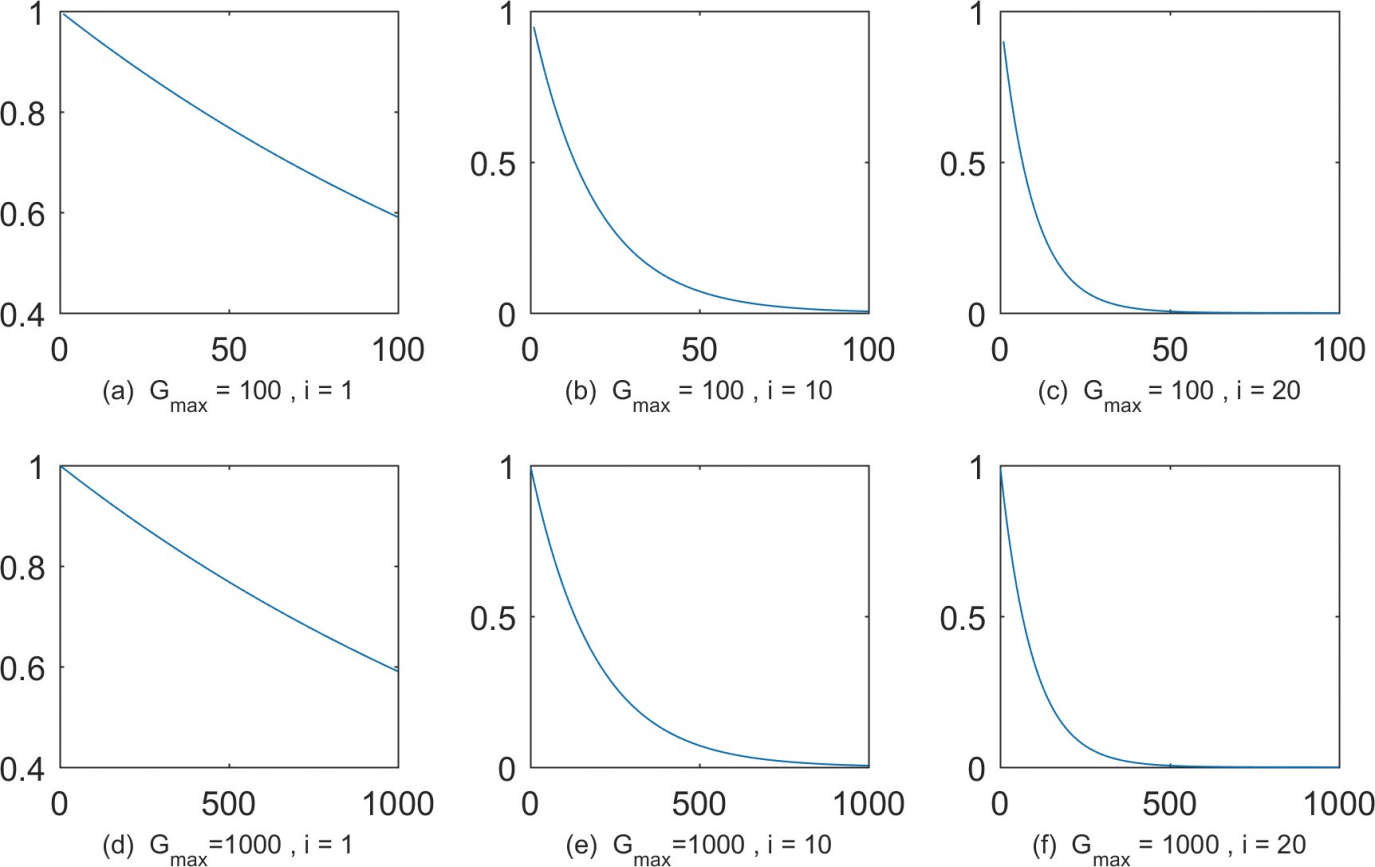
The adaptation curves of the scaling factor with different maximum generation numbers and different individuals. The horizontal axis and the vertical axis are generations and the scaling factor. (a) *G*_*max*_ = 100,*i* = 1; (b) *G*_*max*_ = 100,*i* = 10; (c) *G*_*max*_ = 100,*i* = 20; (d) *G*_*max*_ = 1000,*i* = 1; (e) *G*_*max*_ = 1000,*i* = 10; (f) *G*_*max*_ = 1000,*i* = 20.

Without considering the evolutionary status (set all ${bs}_{i}^{G-1}=1$), the maximum generation number is designed as 100 and 1000 respectively. Taking the 1*st*, 10*th*, 20*th* individuals as examples, the dynamic adaptation curves of the scaling factor *F* are shown in Fig 1.

As *F* approaches zero, the search is conducted over a minimal area around the base vector. Conversely, the larger *F* is, the larger the search area around the base vector is. Observing Fig 1A–1C and Fig 1D–1F from the horizontal direction, it can be concluded that, with the same maximum generation number, the better the individual (the smaller the index number), the lager the corresponding generation number when *F* is close to zero. In other words, the better individual has more global search opportunities. By longitudinal comparison of Fig 1A–1C and Fig 1D–1F, it is clear that, although the maximum generation number is different, the dynamic adaptation curves of scaling factors of individuals with the same index are similar. In other words, for optimization problems with different complexity, the ratios of global and local search are similar. These are precisely in line with our design concepts of *F*.

#### 3.2.2 The crossover probability *CR* adaptation

The crossover probability *CR* indicates the probability of inheriting elements from the mutation individual *V*_*i*_ in the process of constructing the trial individual *U*_*i*_. Generally speaking, the superior individual is closer to the global optimal than the inferior individual [35]. With a smaller *CR*, the offspring individual can obtain more useful information by inheriting more elements from a superior parent individual. Instead, a larger *CR* can generate a more promising offspring individual by accepting more elements from the mutation individual if the parent individual is far away from the optimum. Based on this analysis, *CR* is calculated as follow:

$$CR_{i}=CR_{min}+\frac{i}{NP}\cdot rand_{i}$$ (8)

where *CR*_*min*_ is the minimum *CR*, which is set as 0.1 in this paper. *i* is the ranked index of $X_{i}^{G}$. *NP* denotes the population size, and *rand*_*i*_ denotes a random number uniformly distributed in the interval [0,1].

#### 3.2.3 The weighting factor *W* adaptation

In RHRMDE, looking forward to more local search ability to accelerate convergence, the random elite strategy is proposed with a weighting factor, which is calculated by:

$$W_{i}={\left(1-\frac{G}{G_{max}}\right)}^{2}\cdot \frac{f_{max}-f_{i}}{f_{max}-f_{min}}$$ (9)

where *f*_*min*_, *f*_*max*_, *f*_*i*_ are the fitness value of the best, worst and *i*th individuals in the ranked population. In particular, when *f*_*max*_ is equal to *f*_*min*_, *W*_*i*_ is set to a uniformly distributed random variable within [0,1] to increase diversity. With the deepening of generation, the weighting factor *W*_*i*_ decreases, so that the random elite strategy has a stronger local optimization ability.

**Fig 2:**
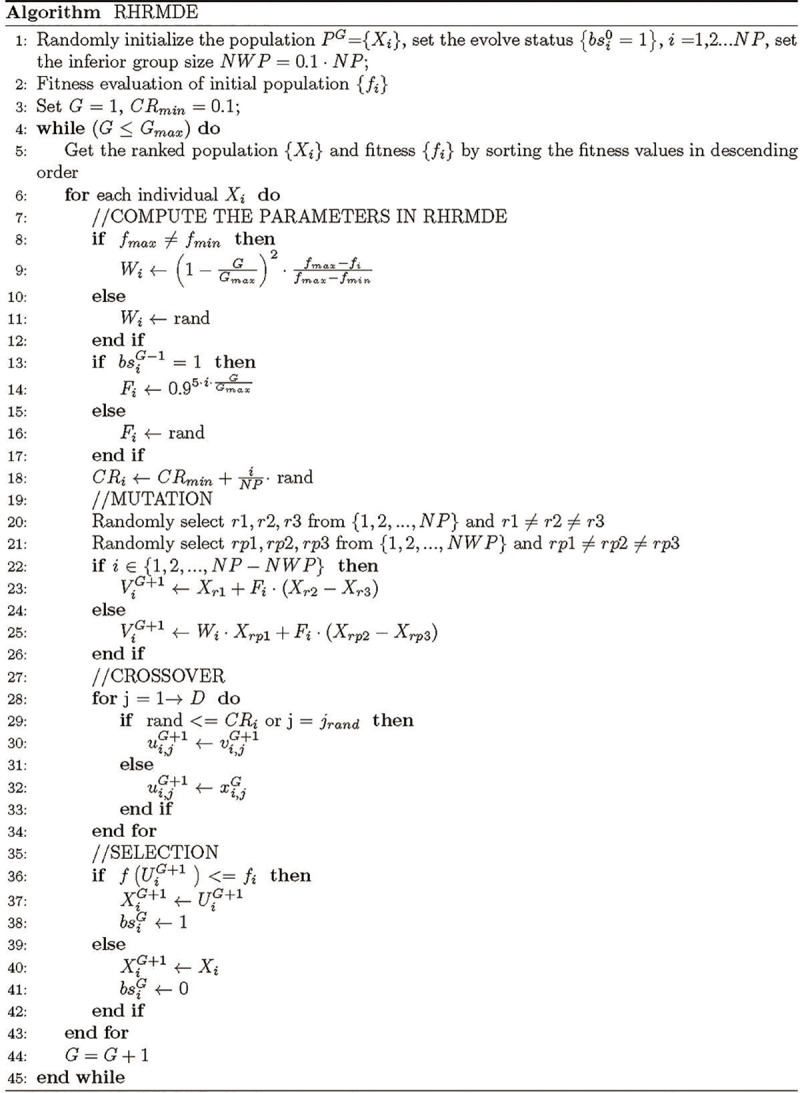
The pseudo-code of RHRMDE.

The pseudo-code for RHRMDE is given in Fig 2.

## 4 Experiments and results analysis

### 4.1 Benchmark test functions

To test the efficiency of RHRMDE, 32 benchmark test functions from literature [42–44] are utilized. All the test functions are treated as black-box problems and tested on 30D,100D, the search domain and the global minimum *f*(*) of these benchmarks can be seen in Table 1. These test functions have many properties, such as unimodality, multimodality, non-separation, separation, shifted, rotated, scalable and non-scalable [21]. In these 32 benchmarks, *f*_1_−*f*_12_ are unimodal functions, *f*_13_ is the step function, *f*_14_ is the noise quartic function, and *f*_15_−*f*_32_ are multimodal functions.

**Table 1:** The benchmark test functions.

Name	Function	Domain	*f*(*)
Sphere	$f_{1}(x)=\sum_{i=1}^{D}{x_{i}}^{2}$	[−100,100]^*D*^	0
Elliptic	$f_{2}(x)=\sum_{i=1}^{D}{\left(10^{6}\right)}^{\frac{i-1}{D-1}}{x_{i}}^{2}$	[−100,100]^*D*^	0
Bent Cigar	$f_{3}(x)={x_{1}}^{2}+10^{6}\sum_{i=2}^{D}{x_{i}}^{2}$	[−100,100]^*D*^	0
Schwefel 1.2	$f_{4}(x)=\sum_{i=1}^{D}{\left(\sum_{j=1}^{i}x_{j}\right)}^{2}$	[−100,100]^*D*^	0
Schwefel 2.22	$f_{5}(x)=\sum_{i=1}^{D}\left|x_{i}\right|+\prod_{i=1}^{D}\left|x_{i}\right|$	[−10,10]^*D*^	0
Schwefel 2.21	$f_{6}(x)=max\{|x_{i}|,1\leq i\leq D\}$	[−100,100]^*D*^	0
Sum of Different Power	$f_{7}(x)=\sum_{i=1}^{D}{\left|x_{i}\right|}^{i+1}$	[−100,100]^*D*^	0
Sum Squares	$f_{8}(x)=\sum_{i=1}^{D}i{x_{i}}^{2}$	[−10,10]^*D*^	0
Discus	$f_{9}(x)=10^{6}{x_{1}}^{2}+\sum_{i=2}^{D}{x_{i}}^{2}$	[−100,100]^*D*^	0
Different Powers	$f_{10}(x)=\sqrt{\sum_{i=1}^{D}{\left|x_{i}\right|}^{2+4\frac{i-1}{D-1}}}$	[−100,100]^*D*^	0
Exponential	$f_{11}(x)=-exp(-0.5\sum_{i=1}^{D}{x_{i}}^{2})$	[−1,1]^*D*^	-1
Zakharov	$f_{12}(x)=\sum_{i=1}^{D}{x_{i}}^{2}+{\left(\sum_{i=1}^{D}0.5x_{i}\right)}^{2}+{\left(\sum_{i=1}^{D}0.5x_{i}\right)}^{4}$	[−5,10]^*D*^	0
Step	$f_{13}(x)=\sum_{i=1}^{D}{\left(\left|x_{i}+0.5\right|\right)}^{2}$	[−100,100]^*D*^	0
Noise quartic	$f_{14}(x)=\sum_{i=1}^{D}i{x_{i}}^{4}+rand[0,1)$	[−1.28,1.28]^*D*^	0
Rosenbrock	$f_{15}(x)=\sum_{i=1}^{D-1}\left[100{∙({x_{i}}^{2}-x_{i+1})}^{2}+{\left(x_{i}-1\right)}^{2}\right]$	[−30,30]^*D*^	0
Griewank	$f_{16}(x)=\sum_{i=1}^{D}\frac{{x_{i}}^{2}}{4000}-\prod_{i=1}^{D}\cos\left(\frac{x_{i}}{\sqrt{i}}\right)+1$	[−600,600]^*D*^	0
Rastrigin	$f_{17}(x)=\sum_{i=1}^{D}\left({x_{i}}^{2}-10\;\cos\left(2\pi x_{i}\right)+10\right)$	[−5.12,5.12]^*D*^	0
Apline	$f_{18}(x)=\sum_{i=1}^{D}\left|x_{i}\sin x_{i}+0.1x_{i}\right|$	[−100,100]^*D*^	0
Bohachevsky_2	$f_{19}(x)=\sum_{i=1}^{D-1}\left[{x_{i}}^{2}+{2x}_{i+1}^{2}-0.3\;\cos\left(3\pi x_{i}\right)\cos\left(3\pi x_{i+1}\right)+0.3\right]$	[−100,100]^*D*^	0
Salomon	$f_{20}(x)=1-\cos\left(2\pi \sqrt{\sum_{i=1}^{D}{x_{i}}^{2}}\right)+0.1\sqrt{\sum_{i=1}^{D}{x_{i}}^{2}}$	[−100,100]^*D*^	0
Scaffer2	$f_{21}(x)=\sum_{i=1}^{D}{\left({x_{i}}^{2}+x_{i+1}^{2}\right)}^{0.25}(\sin\left(50{\left(x_{i}^{2}+x_{i+1}^{2}\right)}^{0.1}\right)+1)\;x_{D+1}=x_{1}$	[−100,100]^*D*^	0
Ackley	$f_{22}(x)=-20exp(-0.2\sqrt{\frac{1}{D}\sum_{i=1}^{D}x_{i}^{2}})-exp(\frac{1}{D}\sum_{i=1}^{D}\cos\left(2\pi x_{i}\right))+20+e$	[−32,32]^*D*^	0
Weierstrass	$f_{23}(x)=\sum_{i=1}^{D}\left(\sum_{k=0}^{k_{max}}\left[a^{k}\;\cos\left(2\pi b^{k}(x_{i}+0.5)\right)\right]\right)-D\sum_{k=0}^{k_{max}}\left[a^{k}\;\cos\left(2\pi b^{k}∙0.5\right)\right]$ *a* =0.5, *b* = 3, *k*_*max*_ = 20	[−0.5,0.5]^*D*^	0
Katsuura	$f_{24}(x)=\frac{10}{D^{2}}\prod_{i=1}^{D}{\left(1+i\sum_{j=1}^{32}\frac{\left|2^{j}x_{i}-round(2^{j}x_{i})\right|}{2^{j}}\right)}^{\frac{10}{D^{1.2}}}-\frac{10}{D^{2}}$	[−100,100]^*D*^	0
HappyCat	$f_{25}(x)={\left|\sum_{i=1}^{D}{x_{i}}^{2}-D\right|}^{1/4}+\left(0.5\sum_{i=1}^{D}{x_{i}}^{2}+\sum_{i=1}^{D}x_{i}\right)/D+0.5$	[−100,100]^*D*^	0
HGBat	$f_{26}(x)={\left|{\left(\sum_{i=1}^{D}{x_{i}}^{2}\right)}^{2}-{\left(\sum_{i=1}^{D}x_{i}\right)}^{2}\right|}^{1/2}+\left(0.5\sum_{i=1}^{D}{x_{i}}^{2}+\sum_{i=1}^{D}x_{i}\right)/D+0.5$	[−100,100]^*D*^	0
Scaffer’s F6	$f_{27}(x)=\sum_{i=1}^{D}\left(0.5+\frac{{\left(\sin\left(\sqrt{{x_{i}}^{2}+x_{i+1}^{2}}\right)\right)}^{2}-0.5}{{\left(1+0.001({x_{i}}^{2}+x_{i+1}^{2})\right)}^{2}}\right)\;x_{D+1}=x_{1}$	[−0.5,0.5]^*D*^	0
Expanded Scaffer	$f_{28}(x)=f_{27}(x_{1},x_{2})+f_{27}(x_{2},x_{3})+\cdots +f_{27}(x_{D-1},x_{D})+f_{27}(x_{D},x_{1})$	[−5,5]^*D*^	0
Griewank+Rosenbrock	$f_{29}(x)=f_{16}(f_{15}(x_{1},x_{2}))+f_{16}(f_{15}(x_{2},x_{3}))+\cdots +f_{16}(f_{15}(x_{D-1},x_{D}))+f_{16}(f_{15}(x_{D},x_{1}))$	[−5.12,5.12]^*D*^	0
NCRastrigin	$f_{30}(x)=\sum_{i=1}^{D}\left[{y_{i}}^{2}-10\;\cos\left(2\pi y_{i}\right)+10\right],y_{i}=\left\{ \begin{array}{l} x_{i}\;,|x_{i}|<0.5\\ \frac{round(2x_{i})}{2},|x_{i}|\geq 0.5 \end{array}\right.$	[−10,10]^*D*^	0
Levy and Montalvo 1	$f_{31}(x)=\frac{\pi }{D}\left\{10{\left(\sin\left(\pi y_{1}\right)\right)}^{2}+\sum_{i=1}^{D-1}{\left(y_{i}-1\right)}^{2}[1+10{\left(\sin\left(\pi y_{i+1}\right)\right)}^{2}]+{\left(y_{D}-1\right)}^{2}\right\}+\sum_{i=1}^{D}u(x_{i},10,100,4)$ $y=1+\frac{1}{4}(x_{i}+1),u(x_{i},a,k,m)=\left\{ \begin{array}{l} k{\left(x_{i}-a\right)}^{m},\;x_{i}>a\\ 0,-a\leq x_{i}\leq a\\ k{\left({-x}_{i}-a\right)}^{m},\;x_{i}<-a \end{array}\right.$	[−10,10]^*D*^	0
Levy and Montalvo 2	$f_{32}(x)=0.1\left\{10{\left(\sin\left(3\pi x_{1}\right)\right)}^{2}+\sum_{i=1}^{D-1}{\left(x_{i}-1\right)}^{2}[1+{\left(\sin\left(3\pi x_{i+1}\right)\right)}^{2}]+{\left(x_{D}-1\right)}^{2}\left[1+{\left(\sin\left(2\pi x_{D}\right)\right)}^{2}\right]\right\}+\sum_{i=1}^{D}u(x_{i},5,100,4)$	[−5,5]^*D*^	0

### 4.2 Computational complexity

The computational complexity of the RHRMDE is mainly composed of the following parts: initialization, fitness evaluation, mutation, crossover and selection, which are represented by Big-O notation respectively as follows: *O*(*NP*∙*D*), *O*(*NP*∙*G*_*max*_), *O*(*NP*∙*D*∙*G*_*max*_), *O*(*NP*∙*D*∙*G*_*max*_), *O*(*NP*∙*G*_*max*_) where *NP* is the population size, *D* denotes the dimension of the specific problem, and *G*_*max*_ indicates the maximum generation number. In addition, the computational complexity of the fitness ranking and individual position updating before each generation of RHRMDE is *O*(*NP*∙*G*_*max*_)+*O*(*NP*∙*D*∙*G*_*max*_). Therefore, the RHRMDE has a computational complexity of *O*(*NP*∙(3∙*D*∙*G*_*max*_+3∙*G*_*max*_+*D*)), which is simply *O*(*NP*∙*D*∙*G*_*max*_).

### 4.3 Sensitivity analysis to the inferior group size *NWP*

**Fig 3:**
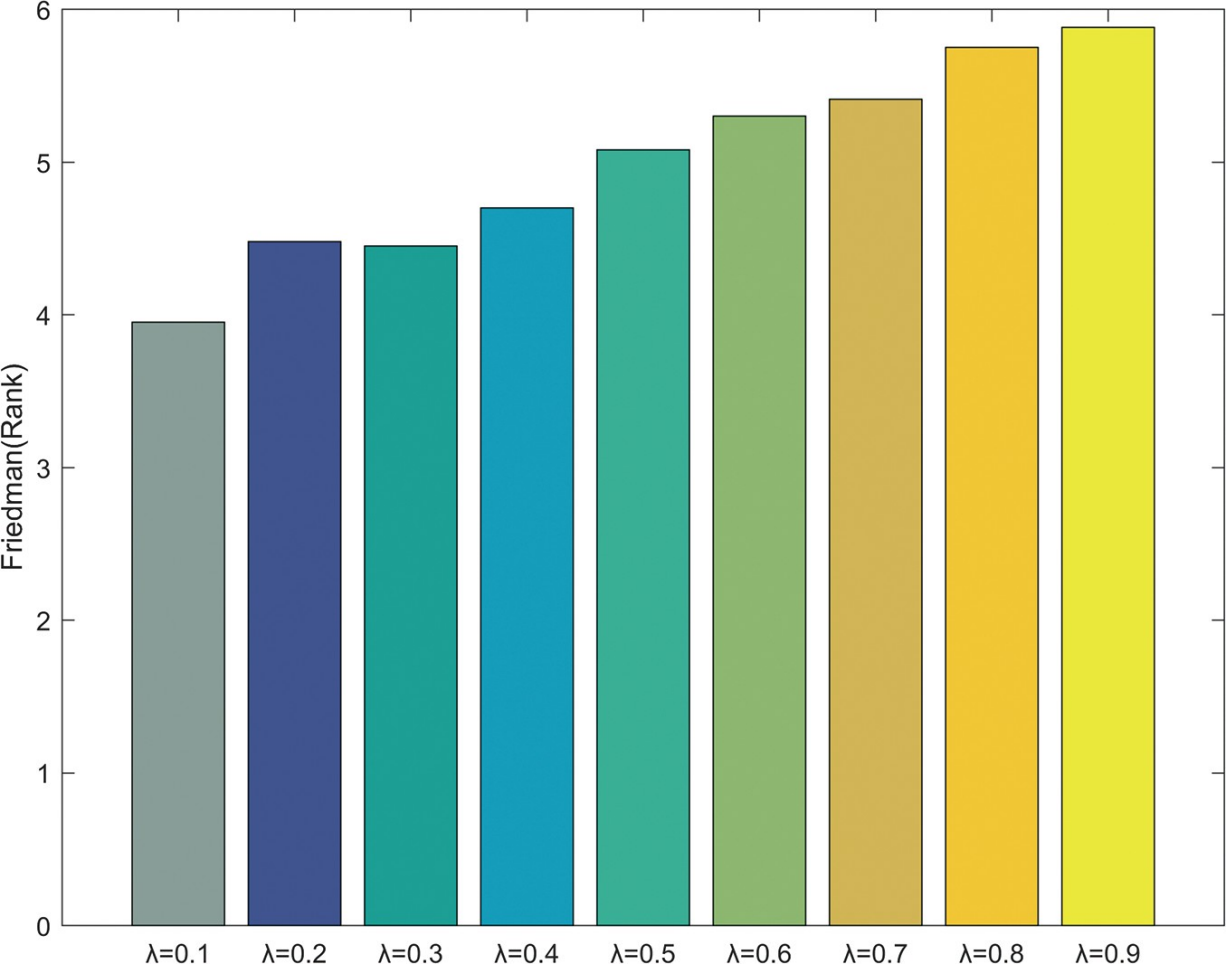
The results of Friedman test with 30 variables over 30 independent runs.

The impact of the inferior group size *NWP* on the performance of RHRMDE is investigated by setting *NWP* = *λ*∙*NP*, and the candidate values for *λ* are 0.1, 0.2, 0.3, 0.4, 0.5, 0.6, 0.7, 0.8, 0.9, respectively. The Friedman test and Wilcoxon’s rank-sum test [45] are conducted for optimization results under different *λ* values, and the test results are shown in Fig 3 and Table 2, respectively.

**Table 2:** The results of Wilcoxon’s rank-sum test over 30 independent runs.

**RHRMDE with *λ* = 0.1 vs.**	***R***^**+**^	***R*−**	***p*−*value***	***α* = 0.05**	***α* = 0.1**
**RHRMDE with *λ* = 0.2**	40	5	7.23e-01	No	No
**RHRMDE with *λ* = 0.3**	39	6	7.23e-01	No	No
**RHRMDE with *λ* = 0.4**	40	5	7.10e-01	No	No
**RHRMDE with *λ* = 0.5**	45	0	6.97e-01	No	No
**RHRMDE with *λ* = 0.6**	45	0	6.97e-01	No	No
**RHRMDE with *λ* = 0.7**	52	3	5.48e-01	No	No
**RHRMDE with *λ* = 0.8**	55	0	5.37e-01	No	No
**RHRMDE with *λ* = 0.9**	55	0	5.37e-01	No	No

*R*^+^ is the rank sum of the first algorithm over the second algorithm, and *R*^−^ is the rank sum of the second algorithm over the first algorithm. The bigger the *R*^+^, the better the first algorithm. Sign “No” indicates that there is no significant performance discrepancy.

From Fig 3, it can be observed that the performance of RHRMDE is best at *λ* = 0.1. From Table 2, it can be seen that RHRMDE is not sensitive to *NWP*. Based on these statistical results, *λ* = 0.1 is considered a more appropriate value. Therefore, *NWP* = 0.1∙*NP* is employed in the following series of experiments.

### 4.4 Parameter settings and involved algorithms

The performance of RHRMDE is compared with five DE-based algorithms like SaDE [29], RcrIJADE [31], MPEDE [36], DPLDE [37], DEPSO [40] and five non-DE-based algorithms like GWO [7], WOA [8], MKE_v3 [9], MPA [10] and EO [12].

For a fair comparison, the maximum generation number *G*_*max*_ = 1000 is used as the stopping criterion for all algorithms, the population size is set *NP* = 100, and each function runs independently for 30 times. The other parameters of the comparative algorithms are consistent with their original literature.

### 4.5 Results analysis

#### 4.5.1 Convergence accuracy analysis

With the same test function, the average of function error value *f*(*X*_*best*_)−*f*(X*) represents the convergence accuracy of the algorithm, where *X*_*best*_ is the best solution found by the algorithm in each run and X* is the real global optimal solution of the test function. Moreover, the Wilcoxon signed-rank test at the 0.05 significance level, the Wilcoxon’s rank-sum test, the Friedman test and Kruskal Wallis test are conducted on the experimental results to obtain a reliable statistic conclusion.

From Tables 3 and 4, the following conclusions can be drawn. (1) RHRMDE obtains global optimal solutions for 30 and 100 dimensional problems of 12 unimodal functions (*f*_1_~*f*_12_) and 12 multimodal functions (*f*_16_~*f*_24_, *f*_27_~*f*_28_, *f*_30_). (2) When *D* = 30, for step function *f*_13_, Rcr-IJADE gets the global optimal solution while RHRMDE performs better than DPLDE, DEPSO, GWO, WOA, MKE_v3 and MPA; for quartic noise function *f*_14_, EO is the best while RHRMDE performs better than SaDE, MPEDE, DPLDE, DEPSO and MKE_v3; for *f*_15_, *f*_25_ and *f*_32_, Rcr-IJADE is the best while RHRMDE performs better than DPLDE, DEPSO and GWO. In addition, RHRMDE also performs better than SaDE on *f*_15_ and better than WOA, MKE_v3 on *f*_32_; for *f*_29_ and *f*_31_, MPEDE is the best while RHRMDE outperforms SaDE, DPLDE, DEPSO, GWO, WOA, EO on *f*_29_ and outperforms DPLDE, DEPSO, GWO, WOA, MKE_v3, MPA on *f*_31_. (3) When *D* = 100, for *f*_13_ and *f*_32_, Rcr-IJADE is the best while RHRMDE outperforms SaDE, DPLDE, DEPSO, GWO,WOA, MKE_v3, MPA, EO on *f*_13_ and outperforms DPLDE, DEPSO, GWO on *f*_32_; for *f*_14_ and *f*_15_, EO is the best, RHRMDE is better than SaDE, Rcr-IJADE, MPEDE, DPLDE, DEPSO, MKE_v3 on *f*_14_, and slightly worse than MPA, EO on *f*_15_; for *f*_25_ and *f*_26_, WOA is the best while RHRMDE outperforms DPLDE, DEPSO on *f*_25_ and outperforms SaDE, Rcr-IJADE, MPEDE; GWO, MKE_v3 on *f*_26_; for *f*_29_, MPEDE is the best while RHRMDE performs better than SaDE, DPLDE, DEPSO, GWO, MKE_v3 and EO; for *f*_31_, RHRMDE obtains the best solution. (4) Contrary to the performance degradation of other algorithms, RHRMDE performs stably and one more optimal solution obtained on high-dimensional problem compared with low-dimensional problem.

**Table 3:** Mean and STD obtained by SaDE, Rcr-IJADE, MPEDE, DPLDE, DEPSO, GWO, WOA, MKE_v3, MPA, EO and RHRMDE on benchmark functions at 30D.

F	SaDE	Rcr-IJADE	MPEDE	DPLDE	DEPSO	GWO	WOA	MKE_v3	MPA	EO	RHRMDE
	Mean ± STD	Mean ± STD	Mean ± STD	Mean ± STD	Mean ± STD	Mean ± STD	Mean ± STD	Mean ± STD	Mean ± STD	Mean ± STD	Mean ± STD
***f***_**1**_	6.95E-24±1.26E-23	5.05E-70±1.68E-69	6.55E-30±3.22E-29	7.87E-261±0.00E+00	1.25E-97±6.84E-97	1.99E-85±3.61E-85	5.63E-190±0.00E+00	1.08E-17±8.45E-18	3.13E-50±3.98E-50	2.31E-122±6.97E-122	**0.00E+00±0.00E+00**
***f***_**2**_	1.32E-20±1.69E-20	2.39E-65±1.15E-64	3.45E-26±1.75E-25	7.06E-236±0.00E+00	1.06E-96±5.40E-96	8.46E-82±1.55E-81	4.11E-183±0.00E+00	1.18E-14±9.75E-15	2.10E-46±2.97E-46	1.48E-119±3.18E-119	**0.00E+00±0.00E+00**
***f***_**3**_	6.61E-18±1.36E-17	1.52E-63±5.84E-63	1.30E-23±5.20E-23	1.27E-191±0.00E+00	2.04E-93±7.05E-93	2.73E-79±9.61E-79	5.47E-183±0.00E+00	8.27E-12±6.61E-12	4.73E-44±1.55E-43	1.65E-116±3.20E-116	**0.00E+00±0.00E+00**
***f***_**4**_	6.01E-01±5.34E-01	5.85E-11±9.78E-11	2.51E-08±7.77E-08	2.88E-173±0.00E+00	8.46E-94±3.28E-93	2.73E-26±1.31E-25	4.73E+03±3.48E+03	1.46E+01±6.56E+00	1.27E-14±5.20E-14	6.69E-39±1.65E-38	**0.00E+00±0.00E+00**
***f***_**5**_	6.51E-14±3.06E-14	2.34E-37±8.13E-37	2.43E-16±4.38E-16	**0.00E+00±0.00E+00**	5.04E-52±1.66E-51	3.12E-49±4.78E-49	1.79E-113±8.86E-113	3.74E-10±2.25E-10	1.33E-27±2.25E-27	1.01E-68±1.60E-68	**0.00E+00±0.00E+00**
***f***_**6**_	1.93E-02±2.31E-02	8.86E-06±1.36E-05	1.91E-07±1.98E-07	2.27E-67±1.24E-66	1.73E-47±7.91E-47	1.38E-21±1.86E-21	1.81E+01±2.69E+01	2.31E-02±1.21E-02	7.34E-20±5.00E-20	3.59E-32±6.72E-32	**0.00E+00±0.00E+00**
***f***_**7**_	4.30E-19±1.51E-18	5.68E-102±2.52E-101	3.53E-54±1.26E-53	**0.00E+00±0.00E+00**	2.76E-114±1.48E-113	2.99E-262±0.00E+00	9.60E-290±0.00E+00	1.05E-37±2.31E-37	3.00E-112±1.64E-111	**0.00E+00±0.00E+00**	**0.00E+00±0.00E+00**
***f***_**8**_	6.85E-25±6.10E-25	1.02E-69±5.43E-69	3.45E-32±1.74E-31	5.84E-238±0.00E+00	3.54E-100±1.90E-99	6.01E-86±2.27E-85	7.68E-192±0.00E+00	1.37E-18±1.51E-18	5.71E-51±1.12E-50	5.54E-124±1.50E-123	**0.00E+00±0.00E+00**
***f***_**9**_	1.84E-23±2.39E-23	6.44E-69±3.12E-68	3.14E-30±1.01E-29	1.08E-290±0.00E+00	6.01E-94±3.29E-93	2.67E-84±6.67E-84	1.77E-186±0.00E+00	2.10E-17±1.93E-17	1.29E-49±1.90E-49	2.02E-121±6.33E-121	**0.00E+00±0.00E+00**
***f***_**10**_	2.12E-12±3.17E-12	4.13E-40±1.70E-39	7.03E-21±2.29E-20	**0.00E+00±0.00E+00**	1.54E-53±8.43E-53	3.47E-65±4.95E-65	2.30E-115±1.25E-114	6.58E-13±8.14E-13	3.48E-37±4.14E-37	1.86E-94±4.80E-94	**0.00E+00±0.00E+00**
***f***_**11**_	0.00E+00±0.00E+00	7.40E-18±2.82E-17	2.59E-17±4.78E-17	**0.00E+00±0.00E+00**	**0.00E+00±0.00E+00**	1.07E-16±2.03E-17	1.11E-17±3.39E-17	7.03E-17±5.44E-17	**0.00E+00±0.00E+00**	**0.00E+00±0.00E+00**	**0.00E+00±0.00E+00**
***f***_**12**_	7.66E-22±1.15E-21	4.91E-61±2.68E-60	1.14E-32±5.47E-32	7.79E-306±0.00E+00	3.01E-103±8.15E-103	7.74E-83±1.89E-82	1.18E-99±4.86E-99	8.21E-17±8.37E-17	1.32E-49±2.39E-49	4.12E-118±1.10E-117	**0.00E+00±0.00E+00**
***f***_**13**_	6.17E-24±6.45E-24	**0.00E+00±0.00E+00**	2.96E-32±7.70E-32	5.83E+00±2.45E-01	2.61E+00±2.96E-01	1.76E-01±2.10E-01	3.64E-04±1.44E-04	8.50E-18±8.59E-18	1.76E-10±8.76E-11	1.80E-21±4.99E-21	6.88E-20±4.21E-20
***f***_**14**_	5.19E-03±2.25E-03	1.09E-03±3.60E-04	3.64E-03±1.31E-03	3.56E-01±2.55E-01	3.10E-01±1.82E-01	2.50E-04±1.17E-04	5.95E-04±6.74E-04	1.20E-02±3.65E-03	3.30E-04±1.96E-04	**2.17E-04±1.19E-04**	1.26E-03±1.09E-03
***f***_**15**_	2.85E+01±1.51E+01	**1.33E-01±7.28E-01**	2.34E+00±2.15E+00	2.89E+01±9.03E-02	2.81E+01±3.07E-01	2.63E+01±5.51E-01	2.60E+01±2.52E-01	2.30E+01±2.33E+01	2.06E+01±5.30E-01	2.32E+01±1.84E-01	2.60E+01±8.80E-02
***f***_**16**_	9.85E-04±3.21E-03	6.57E-04±2.58E-03	2.14E-03±5.12E-03	**0.00E+00±0.00E+00**	2.22E-03±1.21E-02	1.19E-03±3.76E-03	**0.00E+00±0.00E+00**	4.93E-03±7.97E-03	**0.00E+00±0.00E+00**	**0.00E+00±0.00E+00**	**0.00E+00±0.00E+00**
***f***_**17**_	6.82E-01±1.22E+00	0.00E+00±0.00E+00	1.56E-11±8.03E-11	**0.00E+00±0.00E+00**	2.43E-04±1.33E-03	0.00E+00±0.00E+00	**0.00E+00±0.00E+00**	2.66E+01±8.26E+00	**0.00E+00±0.00E+00**	**0.00E+00±0.00E+00**	**0.00E+00±0.00E+00**
***f***_**18**_	7.87E-04±4.27E-04	2.96E-17±7.68E-17	2.20E-07±9.01E-07	5.79E-83±2.94E-82	5.04E-51±2.70E-50	1.14E-05±4.40E-05	2.79E-92±1.53E-91	6.64E+00±6.18E+00	7.32E-27±1.05E-26	1.66E-67±3.65E-67	**0.00E+00±0.00E+00**
***f***_**19**_	6.53E-02±2.55E-01	2.59E-17±5.40E-17	7.96E-02±3.03E-01	**0.00E+00±0.00E+00**	**0.00E+00±0.00E+00**	**0.00E+00±0.00E+00**	**0.00E+00±0.00E+00**	3.53E-16±3.97E-16	**0.00E+00±0.00E+00**	**0.00E+00±0.00E+00**	**0.00E+00±0.00E+00**
***f***_**20**_	2.03E-01±1.83E-02	1.90E-01±3.05E-02	2.40E-01±4.98E-02	**0.00E+00±0.00E+00**	9.99E-02±1.44E-07	1.30E-01±4.66E-02	1.20E-01±7.14E-02	2.13E-01±3.46E-02	9.99E-02±1.79E-17	9.99E-02±0.00E+00	**0.00E+00±0.00E+00**
***f***_**21**_	1.64E+01±2.39E+00	4.47E+00±8.37E-01	2.28E+00±9.39E-01	5.61E-26±1.48E-25	1.58E+00±8.24E+00	4.25E+00±9.78E+00	2.98E+01±1.55E+01	6.07E+00±4.03E+00	1.41E-02±3.06E-02	1.01E-02±2.76E-02	**0.00E+00±0.00E+00**
***f***_**22**_	4.48E-13±3.90E-13	6.51E-15±1.35E-15	6.75E-15±3.14E-15	**0.00E+00±0.00E+00**	**0.00E+00±0.00E+00**	1.09E-14±2.94E-15	3.43E-15±2.38E-15	9.08E-10±5.14E-10	3.32E-15±9.01E-16	3.55E-15±0.00E+00	**0.00E+00±0.00E+00**
***f***_**23**_	7.60E-14±1.61E-13	1.54E-04±7.53E-04	3.19E-02±1.00E-01	**0.00E+00±0.00E+00**	**0.00E+00±0.00E+00**	**0.00E+00±0.00E+00**	**0.00E+00±0.00E+00**	5.00E-02±2.74E-01	**0.00E+00±0.00E+00**	**0.00E+00±0.00E+00**	**0.00E+00±0.00E+00**
***f***_**24**_	1.52E-01±2.06E-02	7.01E-03±7.88E-04	6.94E-04±1.78E-04	1.84E-08±9.98E-08	6.59E-01±9.57E-02	1.61E-01±6.70E-02	**0.00E+00±0.00E+00**	3.19E-02±9.59E-03	**0.00E+00±0.00E+00**	**0.00E+00±0.00E+00**	**0.00E+00±0.00E+00**
***f***_**25**_	3.54E-01±5.99E-02	**1.32E-01±4.53E-02**	2.71E-01±5.23E-02	1.16E+00±1.88E-01	8.64E-01±8.50E-02	5.15E-01±9.03E-02	4.26E-01±1.66E-01	3.41E-01±8.64E-02	4.60E-01±6.09E-02	3.09E-01±6.74E-02	4.76E-01±6.96E-02
***f***_**26**_	5.01E-01±1.60E-01	4.30E-01±1.08E-01	3.94E-01±1.46E-01	5.00E-01±8.93E-04	4.99E-01±2.06E-03	4.23E-01±6.13E-02	**3.31E-01±1.05E-01**	3.73E-01±1.85E-01	4.03E-01±2.76E-02	4.59E-01±4.32E-02	4.98E-01±9.63E-03
***f***_**27**_	**0.00E+00±0.00E+00**	**0.00E+00±0.00E+00**	**0.00E+00±0.00E+00**	**0.00E+00±0.00E+00**	**0.00E+00±0.00E+00**	**0.00E+00±0.00E+00**	**0.00E+00±0.00E+00**	**0.00E+00±0.00E+00**	**0.00E+00±0.00E+00**	**0.00E+00±0.00E+00**	**0.00E+00±0.00E+00**
***f***_**28**_	1.17E+00±1.75E-01	6.76E-01±9.08E-02	3.74E-01±1.67E-01	**0.00E+00±0.00E+00**	4.46E+00±1.25E+00	6.20E-01±4.27E-01	8.85E-01±1.29E+00	6.74E-01±4.08E-01	2.30E-01±2.17E-01	1.40E+00±4.14E-01	**0.00E+00±0.00E+00**
***f***_**29**_	6.12E+00±4.79E-01	2.90E+00±2.53E-01	**2.13E+00±4.00E-01**	1.33E+01±2.41E-01	1.17E+01±2.99E-01	8.84E+00±1.78E+00	8.88E+00±2.76E+00	3.79E+00±1.02E+00	4.45E+00±1.12E+00	9.84E+00±9.69E-01	4.83E+00±8.62E-01
***f***_**30**_	**0.00E+00±0.00E+00**	3.26E-07±1.45E-06	6.46E-04±3.54E-03	7.50E-11±3.92E-10	1.84E+00±1.01E+01	1.13E+00±2.91E+00	**0.00E+00±0.00E+00**	1.44E+01±5.57E+00	**0.00E+00±0.00E+00**	**0.00E+00±0.00E+00**	**0.00E+00±0.00E+00**
***f***_**31**_	1.08E-27±1.28E-27	1.66E-32±3.85E-33	**1.57E-32±5.57E-48**	7.94E-01±6.92E-02	8.37E-02±3.11E-02	1.01E-02±8.22E-03	5.51E-05±3.02E-05	6.68E-21±8.31E-21	1.74E-11±6.33E-12	1.45E-22±5.63E-22	7.20E-22±4.70E-22
***f***_**32**_	5.17E-26±5.49E-26	**1.35E-31±6.68E-47**	4.10E-30±1.46E-29	2.89E+00±5.48E-02	3.37E-01±7.84E-02	1.88E-01±1.37E-01	5.25E-02±5.25E-02	5.55E-03±5.64E-03	1.48E-03±3.84E-03	6.58E-03±2.50E-02	4.25E-20±2.52E-20

Best results are shown in bold.

**Table 4:** Mean and STD obtained by SaDE, Rcr-IJADE, MPEDE, DPLDE, DEPSO, GWO, WOA, MKE_v3, MPA, EO and RHRMDE on benchmark functions at 100D.

F	SaDE	Rcr-IJADE	MPEDE	DPLDE	DEPSO	GWO	WOA	MKE_v3	MPA	EO	RHRMDE
	Mean ± STD	Mean ± STD	Mean ± STD	Mean ± STD	Mean ± STD	Mean ± STD	Mean ± STD	Mean ± STD	Mean ± STD	Mean ± STD	Mean ± STD
***f***_**1**_	3.01E-04±1.75E-04	9.82E-17±3.79E-16	1.21E-09±9.52E-10	5.60E-279±0.00E+00	6.45E-96±3.32E-95	4.47E-41±4.00E-41	6.18E-190±0.00E+00	5.05E-03±2.05E-03	3.34E-42±3.19E-42	1.03E-85±1.68E-85	**0.00E+00±0.00E+00**
***f***_**2**_	7.82E-01±5.29E-01	3.00E-12±7.05E-12	1.14E-04±1.08E-04	9.52E-297±0.00E+00	7.26E-94±3.95E-93	5.78E-38±3.88E-38	3.39E-185±0.00E+00	2.07E+01±8.67E+00	1.45E-38±1.51E-38	1.10E-81±3.58E-81	**0.00E+00±0.00E+00**
***f***_**3**_	3.25E+02±1.72E+02	3.61E-10±1.09E-09	9.98E-03±2.97E-02	3.21E-207±0.00E+00	1.61E-91±7.79E-91	3.22E-35±2.42E-35	1.59E-179±0.00E+00	5.84E+03±3.39E+03	3.92E-36±4.18E-36	8.01E-80±1.15E-79	**0.00E+00±0.00E+00**
***f***_**4**_	1.62E+03±3.29E+02	1.19E+04±3.01E+04	2.90E+02±1.14E+02	1.48E-271±0.00E+00	7.36E-88±3.93E-87	3.91E-03±1.59E-02	4.95E+05±7.61E+04	5.48E+04±9.41E+03	1.45E-05±5.31E-05	3.65E-10±1.09E-09	**0.00E+00±0.00E+00**
***f***_**5**_	2.02E-03±6.06E-03	8.22E-09±1.32E-08	5.12E-03±2.58E-02	**0.00E+00±0.00E+00**	3.04E-49±1.56E-48	1.18E-24±4.14E-25	6.78E-112±1.88E-111	2.77E-02±7.79E-03	1.39E-24±1.75E-24	2.12E-49±1.75E-49	**0.00E+00±0.00E+00**
***f***_**6**_	1.08E+01±1.28E+00	1.72E+01±2.08E+00	9.27E+00±1.08E+00	8.65E-64±4.74E-63	2.78E-44±1.47E-43	1.39E-06±2.22E-06	6.83E+01±3.00E+01	3.86E+01±4.16E+00	7.65E-16±3.70E-16	3.17E-14±5.32E-14	**0.00E+00±0.00E+00**
***f***_**7**_	8.97E+50±2.59E+51	8.95E+22±3.12E+23	1.23E+39±6.76E+39	**0.00E+00±0.00E+00**	6.32E-117±2.02E-116	1.57E-198±0.00E+00	1.58E-281±0.00E+00	5.54E+60±2.87E+61	3.53E-110±1.89E-109	0.00E+00±0.00E+00	**0.00E+00±0.00E+00**
***f***_**8**_	1.19E-04±5.45E-05	5.90E-17±1.10E-16	7.06E-10±5.93E-10	1.76E-275±0.00E+00	1.34E-99±7.01E-99	2.04E-41±1.83E-41	4.56E-187±0.00E+00	2.36E-03±1.35E-03	1.87E-42±2.54E-42	8.02E-86±1.79E-85	**0.00E+00±0.00E+00**
***f***_**9**_	4.41E-04±2.13E-04	1.07E-16±2.12E-16	4.97E-09±6.52E-09	2.12E-318±0.00E+00	2.40E-94±1.20E-93	6.93E-41±6.83E-41	3.52E-185±0.00E+00	9.77E-03±4.57E-03	8.51E-42±1.43E-41	2.72E-85±3.49E-85	**0.00E+00±0.00E+00**
***f***_**10**_	2.86E-01±9.68E-02	1.00E-08±1.38E-08	9.25E-06±5.99E-06	**0.00E+00±0.00E+00**	1.86E-54±7.42E-54	2.08E-30±2.01E-30	1.50E-106±4.33E-106	4.61E-01±1.67E-01	1.76E-29±1.63E-29	1.61E-63±3.10E-63	**0.00E+00±0.00E+00**
***f***_**11**_	1.61E-08±8.62E-09	6.40E-16±3.11E-16	9.49E-14±1.00E-13	**0.00E+00±0.00E+00**	0.00E+00±0.00E+00	2.89E-16±6.25E-17	1.11E-17±3.39E-17	2.81E-07±1.17E-07	**0.00E+00±0.00E+00**	4.07E-17±5.44E-17	**0.00E+00±0.00E+00**
***f***_**12**_	2.17E-04±1.40E-04	2.97E-14±4.99E-14	3.03E-09±2.29E-09	**0.00E+00±0.00E+00**	1.03E-92±4.19E-92	2.33E-40±3.12E-40	7.66E-23±2.02E-22	2.59E-03±9.64E-04	5.35E-41±8.19E-41	2.09E-82±4.59E-82	**0.00E+00±0.00E+00**
***f***_**13**_	3.21E-04±1.63E-04	**2.02E-16±8.34E-16**	1.26E-09±9.18E-10	2.30E+01±3.44E-01	1.84E+01±7.59E-01	6.15E+00±7.02E-01	8.51E-02±2.18E-02	6.11E-03±3.09E-03	2.07E-02±5.40E-02	1.79E-05±9.64E-06	1.38E-06±1.33E-06
***f***_**14**_	1.25E-01±2.54E-02	3.74E-02±9.96E-03	6.67E-02±1.17E-02	3.19E-01±2.31E-01	2.98E-01±2.23E-01	7.96E-04±4.09E-04	5.42E-04±5.52E-04	2.12E-01±5.18E-02	**5.54E-04±2.50E-04**	4.37E-04±2.62E-04	8.34E-04±5.48E-04
***f***_**15**_	4.40E+02±1.07E+02	1.58E+02±4.90E+01	1.74E+02±5.15E+01	9.89E+01±5.34E-02	9.85E+01±2.99E-01	9.70E+01±8.40E-01	9.63E+01±2.33E-01	4.07E+02±1.20E+02	9.33E+01±8.85E-01	**9.31E+01±5.37E-01**	9.56E+01±1.64E-01
***f***_**16**_	5.75E-03±1.50E-02	1.22E-02±2.78E-02	2.46E-03±4.88E-03	**0.00E+00±0.00E+00**	2.68E-04±1.47E-03	5.87E-04±2.24E-03	3.82E-03±1.48E-02	5.75E-03±6.09E-03	**0.00E+00±0.00E+00**	**0.00E+00±0.00E+00**	**0.00E+00±0.00E+00**
***f***_**17**_	2.64E+02±1.48E+01	1.30E+02±7.43E+00	4.62E+01±1.20E+01	**0.00E+00±0.00E+00**	6.63E-02±2.52E-01	1.11E-01±6.08E-01	**0.00E+00±0.00E+00**	2.61E+02±2.69E+01	**0.00E+00±0.00E+00**	**0.00E+00±0.00E+00**	**0.00E+00±0.00E+00**
***f***_**18**_	5.57E+00±5.66E+00	3.00E-03±1.65E-02	2.60E+00±2.00E+00	1.77E-99±9.69E-99	9.85E-48±5.39E-47	2.37E-04±5.41E-04	1.03E-109±5.64E-109	1.23E+02±3.81E+01	5.98E-24±9.37E-24	5.16E-49±4.46E-49	**0.00E+00±0.00E+00**
***f***_**19**_	6.25E+00±2.61E+00	3.83E+00±2.37E+00	4.97E+00±2.61E+00	0.00E+00±0.00E+00	**0.00E+00±0.00E+00**	**0.00E+00±0.00E+00**	**0.00E+00±0.00E+00**	2.24E+00±1.51E+00	**0.00E+00±0.00E+00**	**0.00E+00±0.00E+00**	**0.00E+00±0.00E+00**
***f***_**20**_	1.56E+00±1.75E-01	8.53E-01±1.22E-01	1.35E+00±2.86E-01	1.00E-02±3.05E-02	9.99E-02±1.87E-07	2.40E-01±4.98E-02	1.40E-01±6.74E-02	1.82E+00±2.22E-01	1.80E-01±4.07E-02	1.03E-01±1.83E-02	**0.00E+00±0.00E+00**
***f***_**21**_	1.61E+02±1.54E+01	7.87E+01±7.48E+00	1.57E+01±7.37E+00	4.57E-29±2.42E-28	4.30E-02±1.52E-01	1.83E+01±3.69E+01	1.05E+02±1.31E+02	6.03E+01±1.41E+01	5.28E-04±4.48E-04	1.40E-02±1.14E-02	**0.00E+00±0.00E+00**
***f***_**22**_	1.97E+00±2.57E-01	1.34E+00±4.72E-01	1.81E+00±2.85E-01	0.00E+00±0.00E+00	**0.00E+00±0.00E+00**	4.44E-14±4.73E-15	3.55E-15±2.80E-15	5.65E-01±6.83E-01	3.55E-15±0.00E+00	6.51E-15±1.35E-15	**0.00E+00±0.00E+00**
***f***_**23**_	2.69E+00±1.24E+00	2.79E+00±1.24E+00	1.37E+01±2.76E+00	0.00E+00±0.00E+00	**0.00E+00±0.00E+00**	2.65E-14±1.48E-14	**0.00E+00±0.00E+00**	7.98E+00±3.86E+00	**0.00E+00±0.00E+00**	**0.00E+00±0.00E+00**	**0.00E+00±0.00E+00**
***f***_**24**_	5.51E-01±4.44E-02	1.54E-01±1.30E-02	9.33E-03±1.63E-03	2.43E-10±1.33E-09	2.06E+00±2.02E-01	5.31E-01±1.06E-01	**0.00E+00±0.00E+00**	1.73E-02±5.40E-03	**0.00E+00±0.00E+00**	**0.00E+00±0.00E+00**	**0.00E+00±0.00E+00**
***f***_**25**_	6.09E-01±6.60E-02	5.55E-01±7.40E-02	5.74E-01±5.98E-02	1.47E+00±2.71E-01	1.18E+00±1.15E-01	8.54E-01±7.15E-02	**4.62E-01±1.79E-01**	7.01E-01±9.11E-02	7.78E-01±4.80E-02	7.17E-01±6.90E-02	9.09E-01±4.63E-02
***f***_**26**_	6.10E-01±1.78E-01	6.00E-01±2.29E-01	5.57E-01±1.99E-01	5.00E-01±1.31E-12	5.00E-01±3.87E-05	5.22E-01±1.01E-01	**4.56E-01±1.55E-01**	6.13E-01±2.69E-01	4.82E-01±1.02E-02	4.94E-01±8.69E-03	5.00E-01±5.44E-06
***f***_**27**_	1.63E-08±9.62E-09	2.49E-15±1.81E-15	8.76E-14±1.00E-13	**0.00E+00±0.00E+00**	**0.00E+00±0.00E+00**	**0.00E+00±0.00E+00**	**0.00E+00±0.00E+00**	2.64E-07±9.34E-08	**0.00E+00±0.00E+00**	**0.00E+00±0.00E+00**	**0.00E+00±0.00E+00**
***f***_**28**_	1.58E+01±8.35E-01	8.65E+00±5.59E-01	2.59E+00±8.24E-01	**0.00E+00±0.00E+00**	2.86E+01±1.08E+01	1.35E+00±1.13E+00	1.47E+00±4.12E+00	3.76E+00±1.24E+00	2.76E+00±1.25E+00	4.29E+00±5.62E-01	**0.00E+00±0.00E+00**
***f***_**29**_	5.02E+01±2.29E+00	2.79E+01±2.29E+00	**1.79E+01±2.90E+00**	4.55E+01±2.17E-01	4.43E+01±4.09E-01	4.16E+01±1.63E+00	3.36E+01±1.45E+01	3.97E+01±8.50E+00	3.49E+01±2.28E+00	4.34E+01±9.23E-01	3.77E+01±1.61E+00
***f***_**30**_	1.08E+02±1.45E+01	1.02E+02±5.38E+00	3.82E+01±8.35E+00	**0.00E+00±0.00E+00**	**0.00E+00±0.00E+00**	3.57E+00±4.32E+00	**0.00E+00±0.00E+00**	3.76E+02±5.74E+01	**0.00E+00±0.00E+00**	**0.00E+00±0.00E+00**	**0.00E+00±0.00E+00**
***f***_**31**_	1.04E-03±5.68E-03	3.11E-03±9.49E-03	1.04E-03±5.68E-03	1.06E+00±4.05E-02	4.15E-01±7.78E-02	1.11E-01±2.55E-02	6.71E-04±2.35E-04	5.21E-03±1.18E-02	8.91E-05±1.24E-04	6.58E-05±3.59E-04	**1.84E-09±3.07E-09**
***f***_**32**_	1.03E-02±1.69E-02	**3.32E-03±1.05E-02**	5.18E-03±5.63E-03	9.92E+00±3.29E-02	4.10E+00±6.43E-01	4.39E+00±4.59E-01	2.13E-01±1.09E-01	3.37E-02±5.39E-02	1.51E-01±2.83E-01	3.37E-01±5.80E-01	9.39E-01±6.87E-01

Best results are shown in bold.

**Fig 4:**
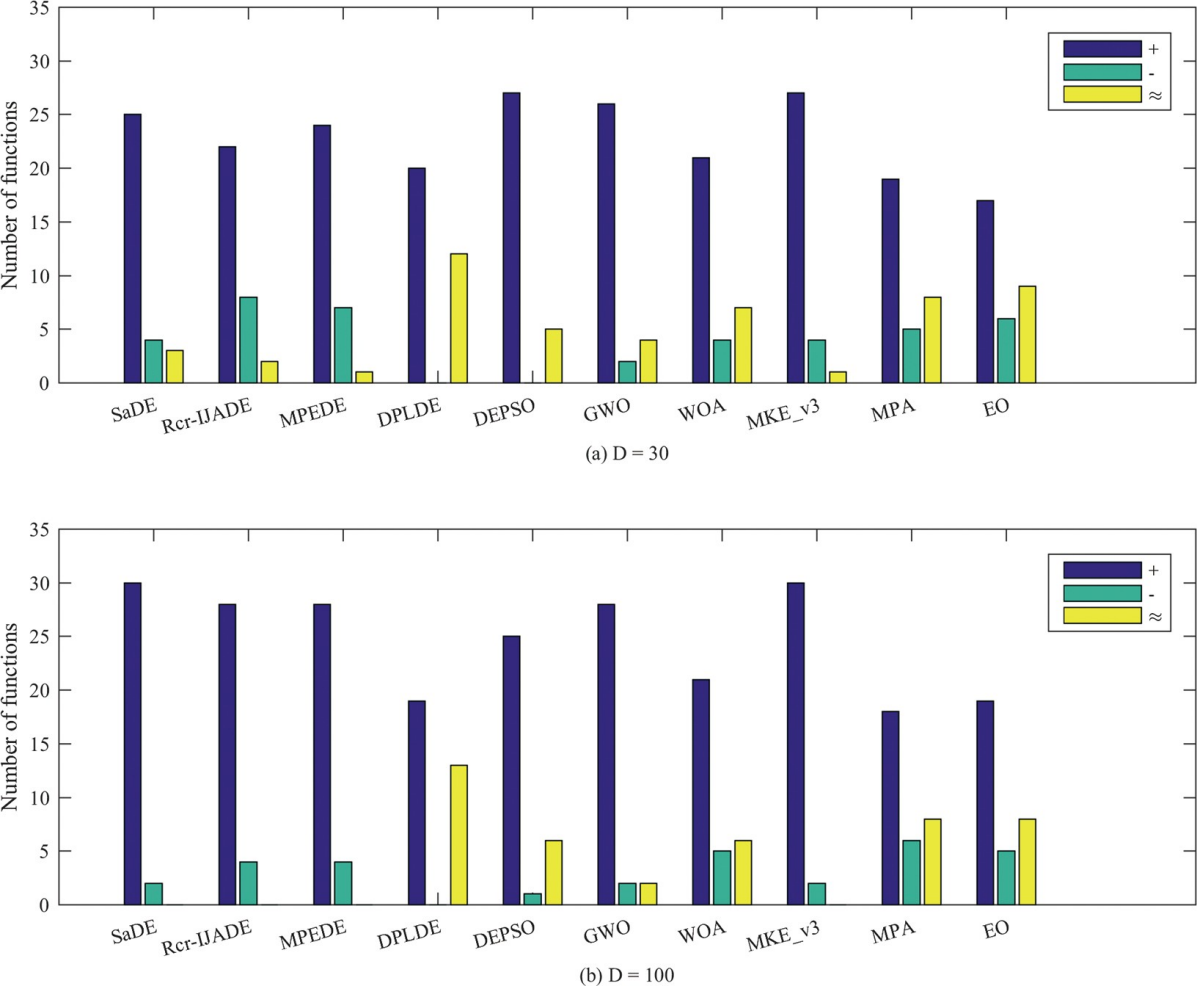
Wilcoxon signed-rank test results at the 0.05 significance level. (a) *D* = 30; (b) *D* = 100.

As shown in Fig 4, RHRMDE performs better than SaDE, Rcr-IJADE, MPEDE, DPLDE, DEPSO, GWO, WOA, MKE_v3, MPA and EO in 25, 22, 24, 20, 27, 26, 21, 27, 19, 17 out of 32 functions at *D* = 30, while 30, 28, 28, 19, 25, 28, 21, 30,18, 19 out of 32 functions at *D* = 100. In contrast, RHRMDE is only worse than SaDE, Rcr-IJADE, MPEDE, DEPSO, GWO, WOA and MKE_v3 in 4, 8, 7, 0, 0, 2, 4, 4, 5, 6 out of 32 functions at *D* = 30, while 2, 4, 4, 0, 1, 2, 5, 2, 6, 5 out of 32 functions at *D* = 100. As a result, RHRMDE is generally superior to SaDE, Rcr-IJADE, MPEDE, DPLDE, DEPSO, GWO, MKE_v3, MPA and EO on these test functions.

According to Table 5, whether *D* equals 30 or 100, it can be observed that RHRMDE gets higher *R*^+^ values than *R*^−^ values for the ten competitors. As can be seen from the computed *p*−*values*, RHRMDE shows a significant improvement in terms of different criteria over SaDE, Rcr-IJADE, MPEDE, DPLDE, DEPSO, GWO, WOA, MKE_v3, MPA and EO at the significance level *α* = 0.05,01. Thus, it can be concluded that RHRMDE is significantly better than its competitors.

**Table 5:** The results of Wilcoxon’s rank-sum test compared with ten algorithms over independent 30 runs.

Comparison	*D* = 30					*D* = 100				
	*R*^+^	*R*^−^	*p*−*value*	*α* = 0.05	*α* = 0.1	*R*^+^	*R*^−^	*p*−*value*	*α* = 0.05	*α* = 0.1
**RHRMDE vs SaDE**	395	40	1.06e-05	Yes	Yes	497	31	2.05e-08	Yes	Yes
**RHRMDE vs Rcr-IJADE**	303	162	5.98e-05	Yes	Yes	454	74	3.20e-07	Yes	Yes
**RHRMDE vs MPEDE**	355	141	8.53e-06	Yes	Yes	459	69	1.32e-07	Yes	Yes
**RHRMDE vs DPLDE**	210	0	1.31e-02	Yes	Yes	190	0	2.64e-02	Yes	Yes
**RHRMDE vs DEPSO**	378	0	1.03e-04	Yes	Yes	338	13	4.27e-04	Yes	Yes
**RHRMDE vs GWO**	372	34	5.85e-05	Yes	Yes	432	33	1.80e-05	Yes	Yes
**RHRMDE vs WOA**	257	68	4.63e-04	Yes	Yes	259	92	4.27e-04	Yes	Yes
**RHRMDE vs MKE_v3**	402	94	2.69e-07	Yes	Yes	498	30	1.27e-08	Yes	Yes
**RHRMDE vs MPA**	198	102	2.08e-03	Yes	Yes	183	117	3.65e-03	Yes	Yes
**RHRMDE vs EO**	176	100	4.94e-03	Yes	Yes	204	96	3.83e-03	Yes	Yes

*R*^+^ is the rank sum of the first algorithm over the second algorithm, and *R*^−^ is the rank sum of the second algorithm over the first algorithm,. The bigger the *R*^+^, the better the first algorithm. Sign “Yes” indicates that the performance of RHRMDE is better than its competitor significantly.

**Fig 5:**
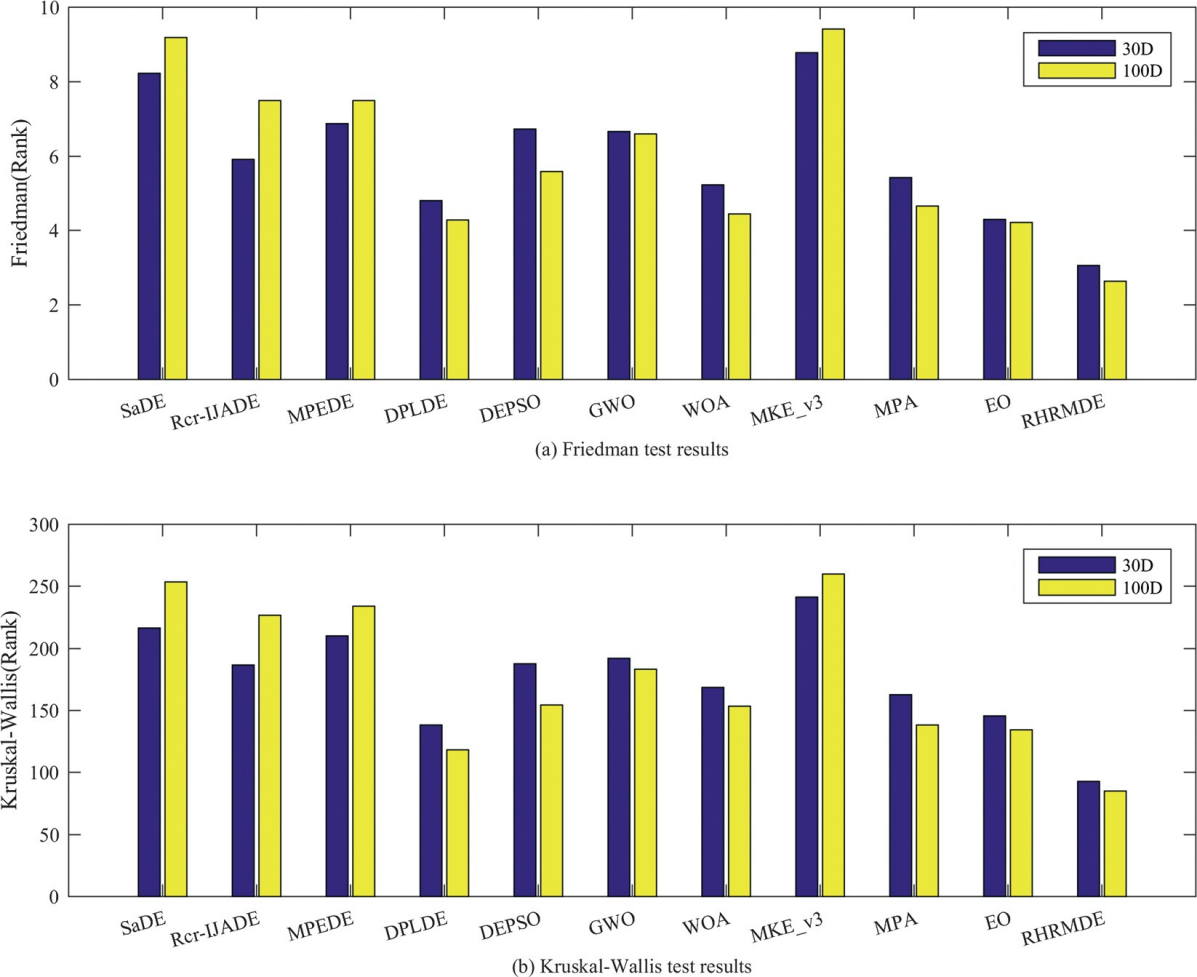
The results of the Friedman test and Kruskal-Wallis test. (a) Friedman test results; (b) Kruskal-Wallis test results.

As can be seen from Fig 5, when *D* = 30,100, both the Friedman test and the Kruskal-Wallis test show that RHRMDE is the best and MKE_v3 is the worst. In addition, EO performs the second best at *D* = 30,100 under the Friedman test, while DPLDE performs the second best at *D* = 30,100 under the Kruskal-Wallis test.

### 4.5.2 Stability analysis

**Fig 6:**
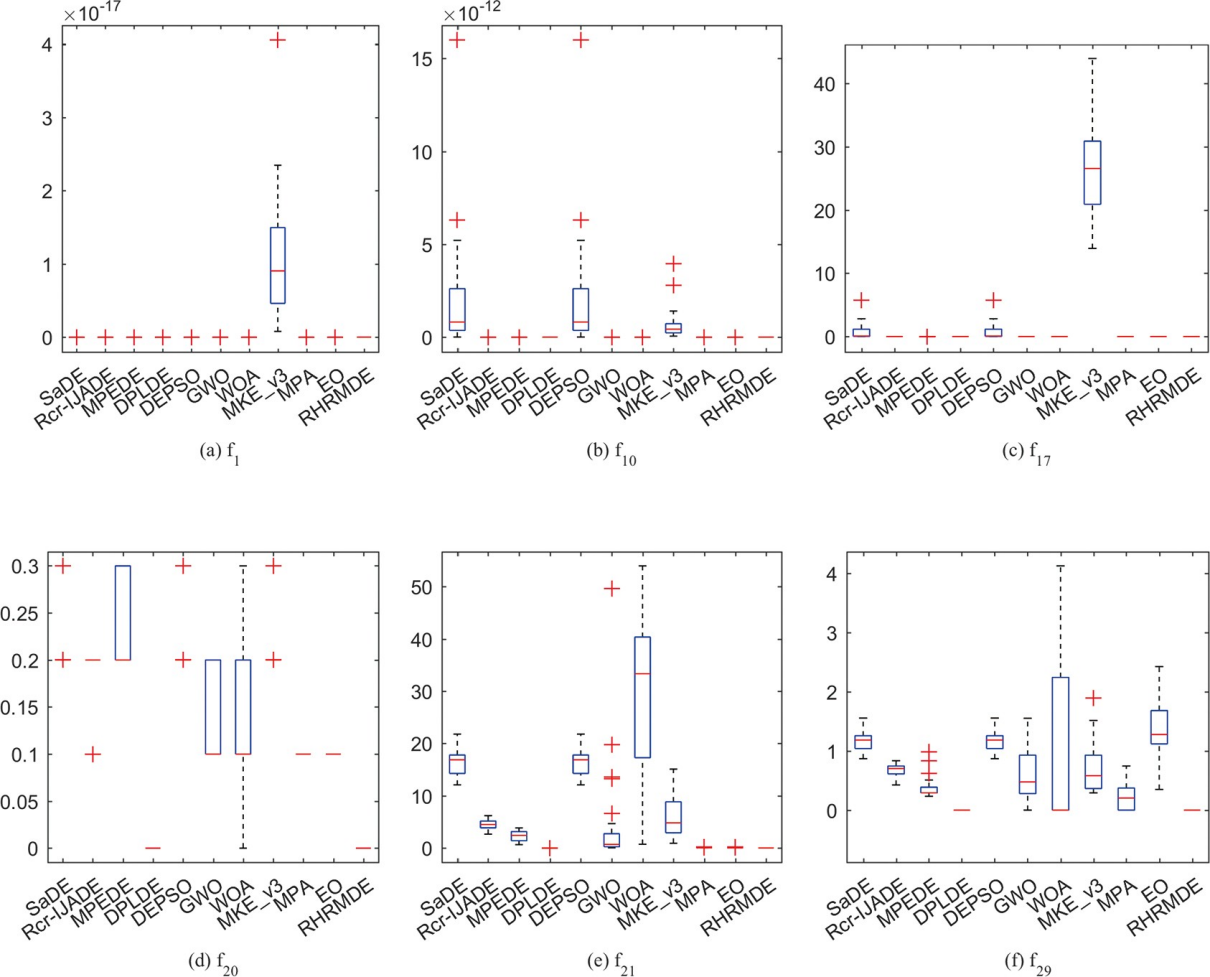
Box plots of the solution error results at *D* = 30 over independent 30 runs. (a) *f*_1_; (b) *f*_10_; (c) *f*_17_; (d) *f*_20_; (e) *f*_21_; (f) *f*_28_.

Due to the stochastic nature of these metaheuristic algorithms, the stability of the proposed algorithm has been analyzed by solution error results over 30 independent runs at *D* = 30. Box plots of solution error results of all compared algorithms on two unimodal and four multimodal functions are shown in Fig 6. It can be seen that RHRMDE has more concentrated solution error values and fewer outliers compared with other algorithms. Therefore, it can be concluded that RHRMDE is superior to other competitors in terms of stability.

### 4.5.3 Convergence speed analysis

**Fig 7:**
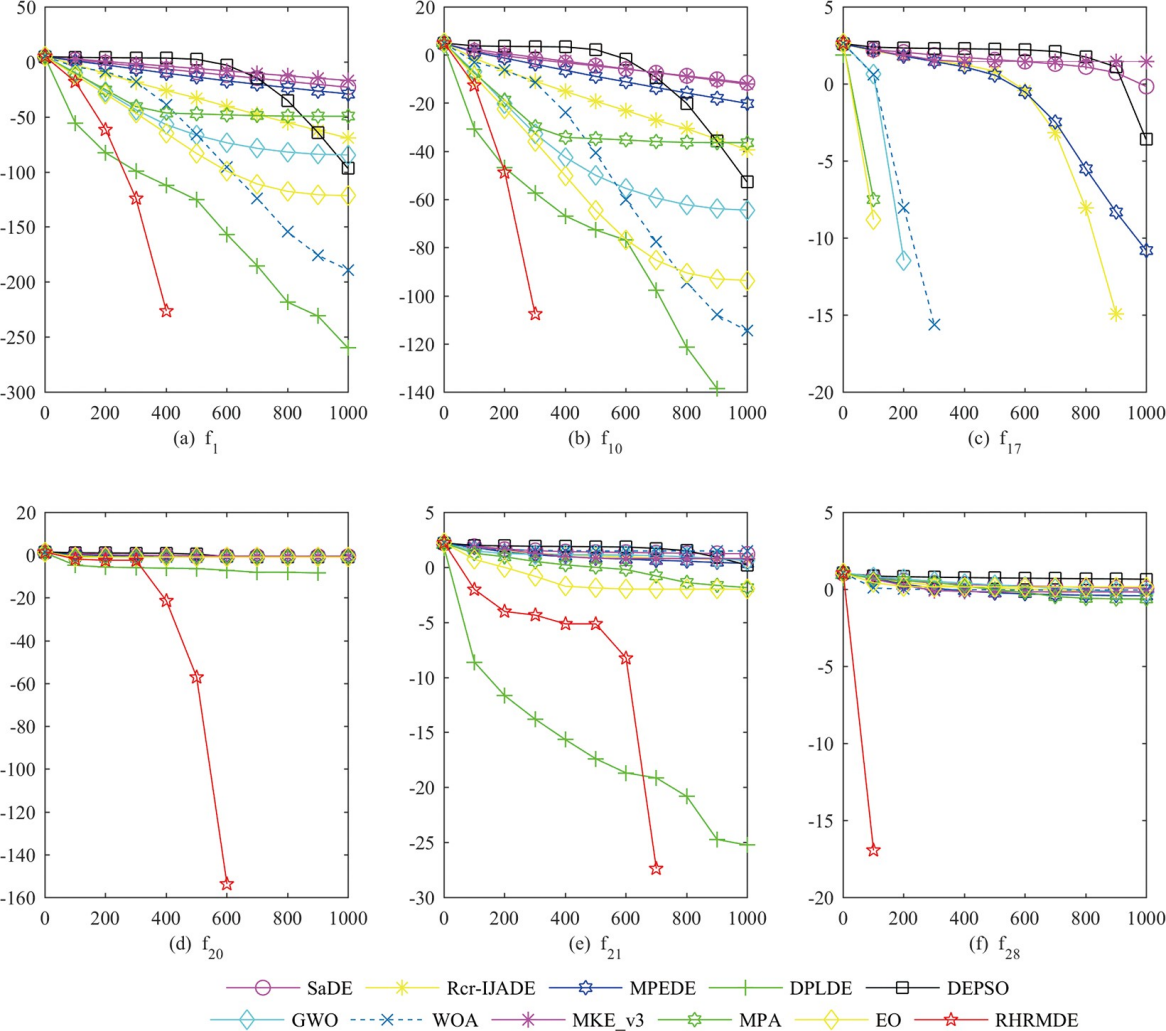
Convergence curves of the mean function error values for six test functions at *D* = 30. The horizontal axis and the vertical axis are generations and the mean function error values over 30 independent runs. a) *f*_1_. (b) *f*_10_. (c) *f*_17_. (d) *f*_20_. (e) *f*_21_. (f) *f*_28_.

In order to compare the convergence speed intuitively, the convergence curves of the mean function error values of all compared algorithms on two unimodal and four multimodal functions at *D* = 30 are plotted in Fig 7. The difference between performance is pronounced in these convergence curves. The steep slope of the RHRMDE curves indicates that RHRMDE converges rapidly and rarely stops before finding the global optimal solution. Due to the introduction of the random global mutation strategy and control parameter adaptation, the population diversity is preserved in the early generations. Therefore, although the convergence speed of RHRMDE is not the fastest in the beginning, it produces the best results in the end. From the perspective of steepness and the final solution, it can be concluded that the convergence speed of RHRMDE is much faster than that of SaDE, Rcr-IJADE, MPEDE, DPLDE, DEPSO, GWO, WOA, MKE_v3, MPA and EO.

### 4.6 Efficiency analysis of control parameter adaptation

The efficiency analysis for control parameter adaptation in RHRMDE is carried out, while the efficiency of hierarchical mutation strategy can be verified by sensitivity analysis to the inferior group size *NWP* as described in section 4.3. Some variants of RHRMDE are listed as follows.

1. To verify the effectiveness of scaling factor *F* adaptation, RHRMDE variants adopt adaptive *CR*, *W* and fixed *F* = 0.3, *F* = 0.7 and *rand* (a uniformly distributed random number in [0, 1]), which are respectively named RHRMDE-1, RHRMDE-2 and RHRMDE-3.
2. To investigate the effectiveness of crossover probability *CR* adaptation, RHRMDE variants with adaptive *F*, *W* and unaltered *CR* = 0.3, 0.7, *rand* are respectively named RHRMDE-4, RHRMDE-5 and RHRMDE-6.
3. To study the effectiveness of weighting factor *W* adaptation, RHRMDE variants with adaptive *F*, *CR* and settled *W* = 0.3, 0.7, *rand* are respectively named RHRMDE-7, RHRMDE-8 and RHRMDE-9.

**Fig 8:**
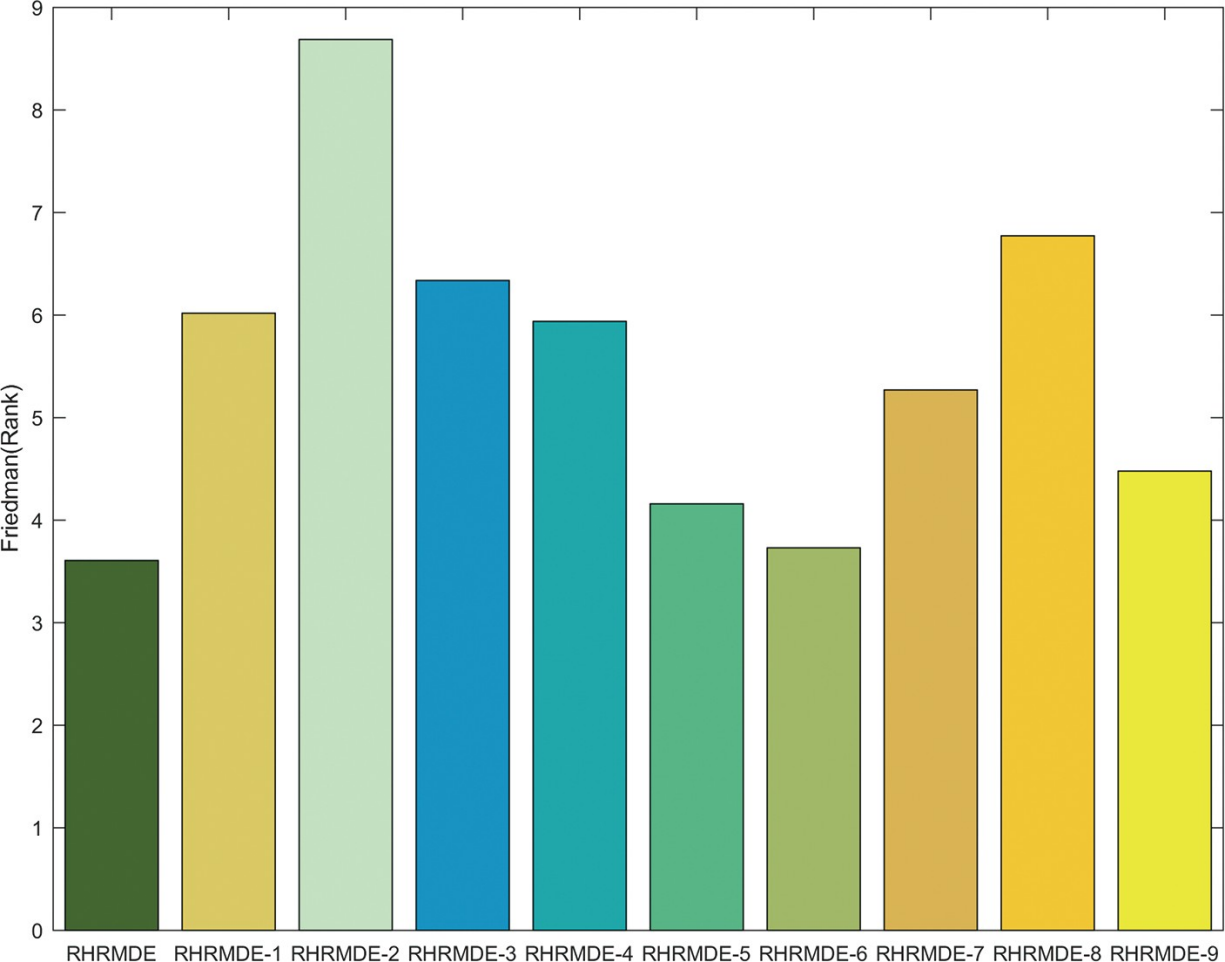
The Friedman test results of proposed RHRMDE and 9 RHRMDE variants with 30 variables over 30 independent runs.

The performance of these different RHRMDE variants is compared in terms of the Friedman test and Wilcoxon’s rank-sum test, the results of which can be seen in Fig 8 and Table 6, respectively. From Fig 8, it can be observed that the proposed RHRMDE, RHRMDE-6, RHRMDE-5 are the best, second best and third best respectively. From Table 6, the proposed RHRMDE outperforms other RHRMDE variants with fixed *F*, *W* and a smaller crossover probability except for RHRMDE-5 with a larger *CR* and RHRMDE-6 with a random *CR*. The above experimental comparisons prove that the control parameter adaptation of RHRMDE is effective and its comprehensive effect is the best. It is worth noting that the contribution of the adaptation of the scaling factor and weighting factor is larger than that of the crossover probability.

**Table 6:** The Wilcoxon’s rank-sum test results of proposed RHRMDE and 9 RHRMDE variants over 30 independent runs.

RHRMDE vs.	*R*^+^	*R*^−^	*p*−*value*	*α* = 0.05	*α* = 0.1
**RHRMDE-1**	226	74	2.18e-03	Yes	Yes
**RHRMDE-2**	361	45	3.20e-05	Yes	Yes
**RHRMDE-3**	175	78	9.34e-03	Yes	Yes
**RHRMDE-4**	222	31	3.84e-03	Yes	Yes
**RHRMDE-5**	48	18	4.49e-01	No	No
**RHRMDE-6**	30	25	6.17e-01	No	No
**RHRMDE-7**	205	48	6.61e-03	Yes	Yes
**RHRMDE-8**	238	87	1.18e-03	Yes	Yes
**RHRMDE-9**	136	95	1.85e-02	Yes	Yes

*R*^+^ is the rank sum of the first algorithm over the second algorithm, and *R*^−^ is the rank sum of the second algorithm over the first algorithm,. The bigger the *R*^+^, the better the first algorithm. Sign “No” indicates that there is no significant performance discrepancy.

### 4.7 Discussion on the comparison results

A series of experiments and comparisons have verified the superior performance of RHRMDE. The reasons are summarized as follows: (1) In the hierarchical mutation mechanism, the random global mutation strategy is conducive to extensive searching in the solution space and maintaining population diversity; the random elite mutation strategy makes full use of randomly selected elite individuals to guide the evolution of inferior individuals, thereby positively affecting the evolution direction of the entire population and accelerating the convergence speed. (2) The scaling factor adaptation considers the complexity differences of various problems and individual differences, and adjusts in combination with the historical evolutionary state. Therefore, the proposed algorithm has different global exploration and local exploitation capabilities for each individual and problem, and can better adapt to various optimization environments. (3) The crossover probability adaptation also takes into account individual differences. When the individual is superior, the crossover probability is more likely to be smaller, so there is a greater likelihood of keeping useful information of the superior individual. (4) Under the influence of fitness value and generations, the weighting factor adaptation improves the local search ability of the proposed algorithm and accelerates the convergence speed of the proposed algorithm.

## 5 Conclusions

A new DE variant algorithm, RHRMDE, is proposed in this paper. In the RHRMDE, the novel hierarchical mutation mechanism based on the non-inferior group and the inferior group is designed to maintain the population diversity and accelerate convergence speed. At the same time, the control parameter adaptation includes historical evolutionary status and individual differences information, considering the complexity differences of various problems, and combining with the hierarchical mutation mechanism to further optimize the algorithm’s balancing ability for exploration and exploitation and speed up the convergence. In addition, the performance of RHRMDE is verified by comparing with five advanced DE variants like SaDE [29], RcrIJADE [31], MPEDE [36], DPLDE [37], DEPSO [40] and five metaheuristic algorithms like GWO [7], WOA [8], MKE_v3 [9], MPA [10] and EO [12] on 32 universal benchmark functions. The experiment results show that: (1) RHRMDE is not sensitive to the inferior group size. (2) In terms of convergence accuracy, stability and convergence speed, RHRMDE performs best for all the unimodal functions and most of the multimodal functions. For the step function, quartic noise function and some multimodal functions, RHRMDE is not the best, which is in line with “No free lunch theorems for optimization” [46]. (3) The adaptive adjustment of the control parameters *F*, *CR* and *W* of RHRMDE is effective, and the integration effect is the best. (4) RHRMDE has the best performance among all the compared algorithms.

As a continuation of this research, the binary and multi-objective version of RHRMDE can be developed. Also, it can be applied to some practical engineering issues, such as airspace planning, flight sequencing, etc.
